# Development of a Multiepitope Vaccine Against SARS-CoV-2: Immunoinformatics Study

**DOI:** 10.2196/36100

**Published:** 2022-07-19

**Authors:** Fatemeh Ghafouri, Reza Ahangari Cohan, Hilda Samimi, Ali Hosseini Rad S M, Mahmood Naderi, Farshid Noorbakhsh, Vahid Haghpanah

**Affiliations:** 1 Department of Biotechnology Faculty of Life Sciences and Biotechnology Shahid Beheshti University Tehran Iran; 2 Department of Nanobiotechnology New Technologies Research Group Pasteur Institute of Iran Tehran Iran; 3 Endocrinology and Metabolism Research Center Endocrinology and Metabolism Clinical Sciences Institute Tehran University of Medical Sciences Tehran Iran; 4 Department of Microbiology and Immunology University of Otago Otago New Zealand; 5 Digestive Diseases Research Center Digestive Diseases Research Institute Tehran University of Medical Sciences Tehran Iran; 6 Department of Immunology School of Medicine Tehran University of Medical Sciences Tehran Iran; 7 Personalized Medicine Research Center Endocrinology and Metabolism Clinical Sciences Institute Tehran University of Medical Sciences Tehran Iran

**Keywords:** SARS-CoV-2, envelope protein, spike protein, COVID-19 vaccine, bioinformatics, COVID-19, informatics, immunoinformatics, computational model, vaccine design, pandemic

## Abstract

**Background:**

Since the first appearance of SARS-CoV-2 in China in December 2019, the world witnessed the emergence of the SARS-CoV-2 outbreak. Due to the high transmissibility rate of the virus, there is an urgent need to design and develop vaccines against SARS-CoV-2 to prevent more cases affected by the virus.

**Objective:**

A computational approach is proposed for vaccine design against the SARS-CoV-2 spike (S) protein, as the key target for neutralizing antibodies, and envelope (E) protein, which contains a conserved sequence feature.

**Methods:**

We used previously reported epitopes of S protein detected experimentally and further identified a collection of predicted B-cell and major histocompatibility (MHC) class II–restricted T-cell epitopes derived from E proteins with an identical match to SARS-CoV-2 E protein.

**Results:**

The in silico design of our candidate vaccine against the S and E proteins of SARS-CoV-2 demonstrated a high affinity to MHC class II molecules and effective results in immune response simulations.

**Conclusions:**

Based on the results of this study, the multiepitope vaccine designed against the S and E proteins of SARS-CoV-2 may be considered as a new, safe, and efficient approach to combatting the COVID-19 pandemic.

## Introduction

The recent outbreak of the new virus in Wuhan City, China, contributed to the discovery of a new coronavirus strain, labeled SARS-CoV-2, of the Coronaviridae family. This virus has caused severe damage and anxiety, leading to the loss of myriad individuals, impacting more than 535,863,950 people to date. SARS-CoV-2 causes the disease named COVID-19, which is associated with symptoms such as a flu-like illness, acute respiratory distress syndrome, and clinical or radiological evidence of pneumonia in individuals needing hospitalization [1]. Patients diagnosed with COVID-19 are reported to have high levels of interleukin (IL)1β, interferon (IFN)γ, interferon-inducible protein 10 (IP10), and monocyte chemoattractant protein 1 (MCP1), likely leading to activated T helper-1 cell responses. In comparison, patients requiring intensive care unit admission had higher concentrations of granulocyte-colony stimulating factor, IP10, MCP1, MIP1A, and tumor necrosis factor-α than those not requiring intensive care, suggesting a possible correlation of cytokine storm and disease intensity. Nonetheless, SARS-CoV-2 infection also resulted in the enhanced production of T helper-2 cell cytokines such as IL4 and IL10, which inhibit inflammation that varies from that induced by SARS-CoV infection [2]. The persistent rise in patients and the high contagious rate of SARS-CoV-2 infection illustrate the immediate need to develop a safe and effective vaccine.

Vaccines are mostly comprised of whole pathogens, either destroyed or attenuated. However, it may be beneficial to use protein vaccines that are capable of generating an immune response against a specific pathogen. Epitope-based vaccines (EVs) utilize immunogenic proteins (epitopes) to induce an immune response. The performance of an EV is calculated by the number of epitopes to be used as the foundation. Nevertheless, the experimental identification of candidate epitopes is costly in terms of both time and money. Moreover, different immunological requirements need to be considered for the final choice of epitopes [3].

**Figure 1:**
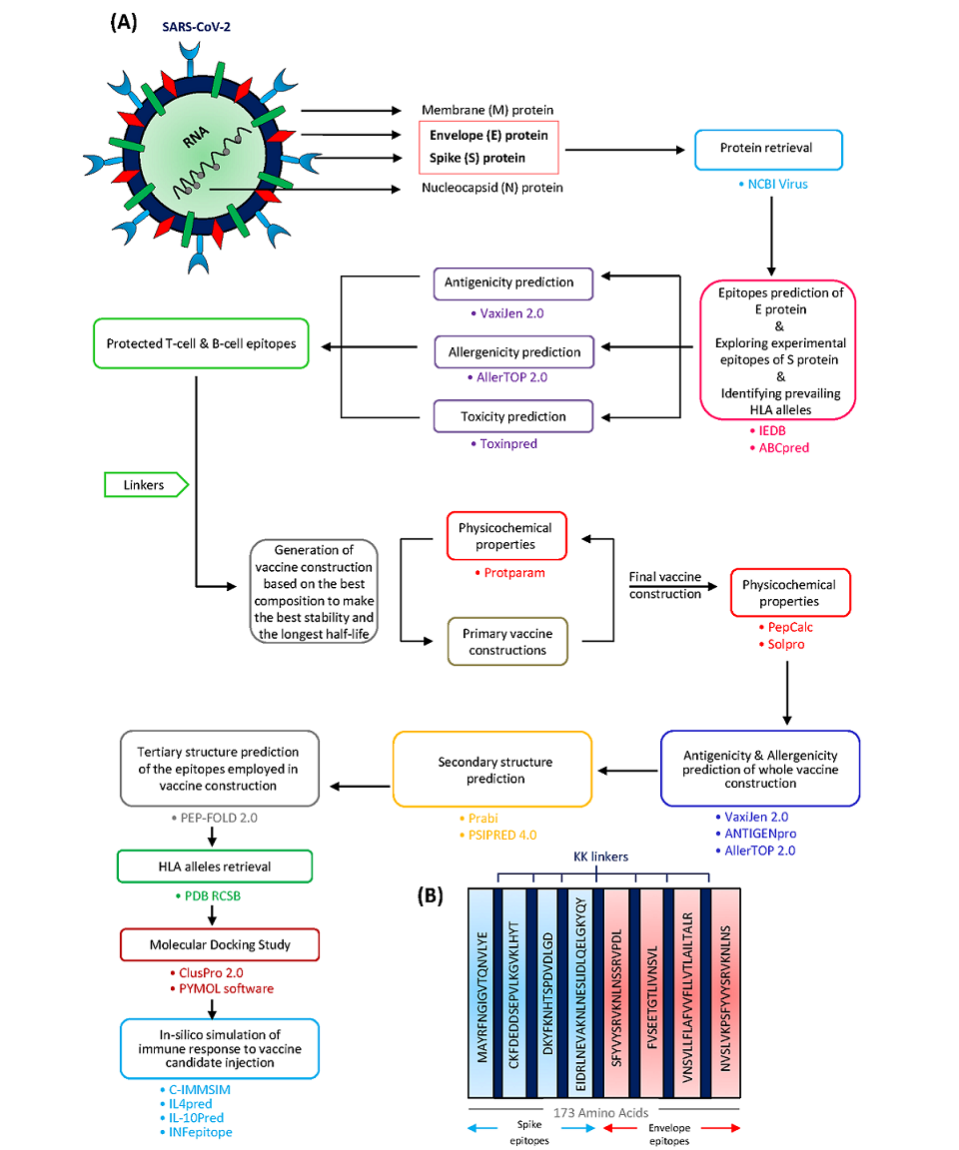
Schematic of the overall study design for development of a SARS-CoV-2 multiepitope vaccine. (A) Study workflow of the in silico design of a multiepitope vaccine against the envelope (E) protein of SARS-CoV-2. (B) Overlaps of 21 selected epitopes merged showing the final construct consisting of 8 epitopes. HLA: human leukocyte antigen; NCBI: National Center for Biotechnology Information.

The properties of coronaviruses can be determined by electron microscopy. Coronaviruses are enveloped viruses with single-stranded positive-sense RNA. The coronavirus genome size varies from 26 to 32 kb [4]. Like all coronaviruses, SARS-CoV-2 comprises four viral proteins, namely spike (S) protein, a type of glycoprotein; membrane (M) protein, covering the membrane; envelope (E) protein, a strongly hydrophobic protein that covers the entire coronavirus structure; and nucleocapsid (N) protein, a structural protein that suppresses RNA interference to overcome the host defense response [5,6] (Figure 1A). Such accessory proteins are not only essential for virion assembly but might also play additional roles in disrupting the host immune responses to promote viral replication [7]. SARS-CoV-2 requires the S glycoprotein, as the key target for neutralizing antibodies, to bind to the receptor and facilitate membrane fusion and virus entry. Every trimeric S protein monomer is roughly 180 kDa in size and comprises two subunits, S1 and S2, mediating binding and membrane fusion, respectively [8]. Therefore, S protein, but not other structural proteins, is the main antigen that causes the production of defensive neutralizing antibodies that stop viruses from attaching to their specific receptor, thereby preventing viral infection [9,10]. The S and M structural proteins have also been shown to have substantial mutational modifications, whereas the E and N proteins are highly conserved (Figure 1A), indicating differential selection pressures imposed on SARS-CoV-2 during evolution [11]. E protein is a small intrinsic membrane protein that is actively engaged in several stages of the life cycle of the virus, such as assembling, propagation, enveloping, and pathogenesis [12]. This protein also slows the transport of proteins through the secretive pathway by adjusting the concentrations of Ca^2+^ and H^+^ in the Golgi and endoplasmic reticulum compartments, which has been suggested as a mechanism for immune avoidance [13].

In this study, the S and E protein sequences were collected from a protein database and analyzed with various bioinformatics tools to identify protective epitopes. The toxicity of whole E protein as a second antigen was analyzed, and toxic epitopes were identified. The predicted B-cell and major histocompatibility complex (MHC) class II–restricted T-cell epitopes were checked in terms of not coinciding with these regions. The presence of less toxic epitopes in E protein in comparison with S protein served as a motivation to design an effective vaccine against these two antigens.

In particular, we sought to design a vaccine against the two structural antigens, S and E proteins, without using built-in adjuvants to obtain a vaccine that could be effective against all current and potential mutations of SARS-CoV-2, along with the advantage of a low molecular weight to avoid the complexity of future manufacturing. Since E protein is more highly conserved and the candidate vaccine showed all of the desired properties in simulations without using adjuvants, the study goals were achieved.

## Methods

### Protein Sequence Retrieval

Based on the vaxquery database [14], the S and E proteins of SARS-CoV-2 were selected as targets for vaccine design because there are already vaccines in development or produced based on these proteins. The amino acid sequences of the E and S proteins of SARS-CoV-2 were collected from the National Center for Biotechnology Information (NCBI) virus database [15] with accession number QHD43418 and QHR63280, respectively.

### B-Cell Epitopes Prediction

Prediction methods are both time- and cost-effective, and are reliable approaches for predicting linear B-cell epitopes as the first step in the genome-wide quest for identifying B-cell antigens in a pathogenic organism [16]. The ABCpred database [17] includes the full-length E protein sequence for the prediction of linear B-cell epitopes. In this study, we set the “threshold” to 0.51 (default value), “length” to 16 (default value), and “overlapping filter” to “NO.” ABCpred uses a machine-learning methodology that requires fixed-length patterns for training or research, and the B-cell epitopes range from 5 to 30 residues in length. To overcome this issue, the server sought to create data sets of fixed-length patterns from B-cell epitopes by removing or linking residues to terminals. With a single hidden layer, the ABCpred server employs a partly recurrent neural network (Jordan network). The networks contain a single hidden layer with 35 residues and selectable window lengths of 10, 12, 14, 16, 18, and 20. The result is a single binary value that is either 1 or 0 (epitope or nonepitope).

The performance of prediction algorithms was evaluated using three parameters [17,18]: sensitivity, specificity, and accuracy. Sensitivity was calculated as the percentage of epitopes correctly identified as epitopes with the formula (TP/[TP+FN])×100, where TP and FN are the numbers of true positives and false negatives, respectively. Specificity was calculated as the percentage of correctly predicted nonepitopes with the formula (TN/[TN+FP])×100, where TN and FP are the numbers of true negatives and false positives, respectively. Accuracy is calculated as the total number of correct predictions (which includes both TP and TN) divided by the total number of forecasts made, multiplied by 100.

The Immune Epitope Database (IEDB) [19] was utilized to browse the available experimental B-cell assays on S protein of SARS-CoV-2. IEDB documents experimental evidence on antibody and T-cell epitopes examined in humans, nonhuman primates, and other animal species in the sense of infectious diseases, allergy, autoimmunity, and transplantation. We used the following parameters for browsing S epitopes: “Epitope” was set to “Linear peptide,” “Organism” was set to “SARS-CoV,” “Antigen” was set to “Spike glycoprotein,” “Assay” was set to “T cell” and “B Cell,” “MHC restriction” was set to “Class II,” and “Host” was set to “Human.”

For the B-cell epitope prediction of E protein using IEDB, we used the Bepipred Linear Epitope Prediction 2.0 service, which is based on a random forest algorithm trained on epitopes and nonepitope amino acids obtained from reported crystal structures to predict B-cell epitopes from a protein sequence, followed by sequential prediction smoothing [20].

### T-Cell Epitopes Prediction

T-cell epitopes are a group of proteins that can be detected by T-cell receptors after a given antigen has been processed intracellularly and attached to at least one MHC molecule, which are then expressed on the surface of antigen-presenting cells (APCs) as an MHC-protein complex. For entities that have at least one MHC molecule with strong affinity for binding to allergenic amino acid sequences from an allergen, the T-cell clones that can detect this MHC-protein complex are genetically susceptible to allergic reactions to this allergen. This concept can be investigated in silico by employing advanced statistical and mathematical methods [21]. The helper T lymphocyte (HTL) epitopes of E protein were predicted by the IEDB database [19]. For T-cell MHC class II epitope prediction by IEDB, the “Prediction method” was set to “IEDB recommended 2.22,” “species/locus” was set to “human”/“HLA-DRA-DBR1*01:01” and “HLA-DPB1*01:02,” and “length” was left as the default setting. IEDB recommends using the consensus method, which compares a variety of methods to predict MHC class II epitopes, including a consensus approach combining NN-align, SMM-align, and combinatorial library methods [19]. The other tool that is available on the IEDB can browse the experimental HTL epitopes through the library based on a relevant antigen [19].

### Antigenicity, Allergenicity, and Toxicity Prediction

VaxiJen v2.0 with a threshold of 0.4 was used to predict the antigenicity of both B-cell and T-cell epitopes. VaxiJen is the first alignment-independent antigen predictor server, which was developed to achieve the categorization of antigens solely based on the physicochemical properties of proteins without recourse to sequence alignment. The system can be used either on its own or in conjunction with alignment-based prediction methods [22]. The methodology of this server is based on z descriptors, autocross covariance (ACC) preprocessing, discriminant analysis by partial least squares, and sequence similarity of the training set [23]. The z descriptors reflect the most critical physicochemical features for antigen recognition, including z1, z2, and z3 descriptors to describe the protein sequences. The hydrophobicity of amino acids is represented by the first principal component (z1), their size is represented by the second component (z2), and their polarity is represented by the third component (z3). The auto covariance Ajj(lag) is represented by Equation (1) [22]:


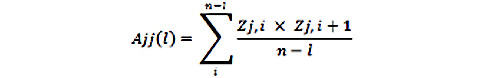
 (1)

The z-scales are calculated using index *j* (*j*=1, 2, 3), *n* (number of amino acids in a sequence), *I* (amino acid position; *I*=1, 2,...n), and *l* (lag) (*l*=1, 2,...L). A small range of lags (L=1,2,3,4,5) was employed to explore the effect of near amino acid proximity on protein antigenicity. Cross covariances Cjk(lag) between two distinct z-scales, *j* and *k*, were calculated with Equation (2):


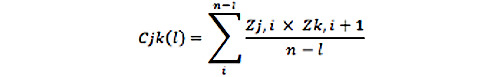
 (2)

VaxiJen v2.0 was used to estimate the antigenicity of the whole-protein chimera. Based on this server, the antigenicity score of the final protein was 0.5830 (Probable ANTIGEN) with a threshold of 0.4. Likewise, ANTIGENpro [24] was utilized to predict the antigenicity of the protein chimera. ANTIGENpro is an alignment-free, sequence-based, and pathogen-independent protein antigenicity predictor. ANTIGENpro is the first indicator of protein antigenicity that is trained to employ reactivity data from the protein microarray analysis of five pathogens.

AllerTOP v2.0 was used to predict the allergenicity of both B-cell and HTL epitopes. Protein sequences are sent to this server in simple text. The results page then provides the identity of an allergen as “probable allergen” or “probable nonallergen.” The whole-protein chimera was predicted as a “probable nonallergen” using this tool [20]. This server was chosen because of its high sensitivity (94%) and higher rate of accurate prediction (94%-100%) in comparison to other similar servers to predict allergenicity [20]. Similar to VaxiJen, this database analyzes the presentation of protein sequences by z-descriptors and ACC transformation [17,18].

ToxinPred [25] with a protein fragment length of 10 was used to predict the toxicity of both B-cell and HTL epitopes. ToxinPred is a computational tool that was built to anticipate and design toxic versus nontoxic proteins. The primary data set used for this approach is comprised of 1805 toxic proteins (≤35 residues). This server also was used to predict the toxicity of the whole-protein chimera and no fragment was predicted as a toxin.

### Construction of the Chimeric Protein

Selected B-cell and HTL epitopes were used to construct the protein chimera as a multiepitope vaccine. Overlaps of B-cell and HTL epitopes were merged. Bilysine (KK) linkers, as flexible linkers, were used to connect the epitopes. The KK linker was implanted between separate epitopes to maintain their independent immunological functions (Figure 1B). KK is the target sequence of cathepsin B, which is one of the essential antigen-processing proteases in MHC class II antigen presentation [26].

### Amino Acid Composition, Physicochemical Properties, and Solubility Prediction

The Protparam database [27] was used to calculate and predict the molecular weight, isoelectric point (pI), in vivo and in vitro half-life, instability index II, and grand average of hydropathicity (GRAVY). ProtParam from the ExPASy server is a reliable algorithm to compute physicochemical properties. However, it uses a single sequence per analysis through the interface. The instability index is calculated using weight values, as shown in Equation (3) [28]:


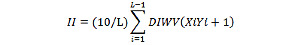
 (3)

where L is the length of the sequence and DIWV(x[*i*]x[*i*+1]) is the instability weight value for the dipeptide starting in position *i*. A protein with an instability index less than 40 is anticipated to be stable, whereas one with an index greater than 40 is predicted to be unstable.

The relative volume occupied by aliphatic side chains (alanine, valine, isoleucine, and leucine) is known as the aliphatic index, which is considered a potentially beneficial element in the enhancement of globular protein thermostability. The aliphatic index is calculated by the formula X(Ala)+*a*X(Val)+*b*(X[Ile]+X[Leu]) [29], where X(Ala), X(Val), X(Ile), and X(Leu) represent the mole percent (100×mole fraction) of alanine, valine, isoleucine, and leucine, respectively, and the coefficients *a* and *b* are the relative volumes of the valine side chain (*a*=2.9) and Leu/Ile side chains (*b*=3.9) to the side chain of alanine.

The SOLpro program from ANTIGENpro [24] was used to predict the solubility of protein chimera upon overexpression. SOLpro predicts the tendency of a protein to be soluble when overexpressed in *Escherichia coli* using a two-stage support vector machine model based on multiple representations of the primary sequence.

The PepCalc server [30] was used to predict the solubility of the final protein, which provides only a very rough estimation of water solubility.

### Secondary Structure Prediction

The Prabi server [31] was used to predict the secondary structure of the final sequence of the protein chimera. All PRABI components provide services in their various areas of expertise (eg, molecular, phylogeny, genomics, transcriptomics, proteomics, protein structure, and medical biostatistics). “GOR IV” was selected as the secondary structure prediction method. The program outputs two files: one with the sequence and anticipated secondary structure in rows (H=helix, E=extended or beta strand, and C=coil), and the other with the probability values for each secondary structure at each amino acid position (H=helix, E=extended or beta strand, and C=coil) [32].

The PSIPRED 4.0 [33] server was also used to predict the secondary structure, which provides more details of residues’ configurations. This is a very simple system of secondary prediction based on a simple neural network evaluation of PSI-BLAST–generated profiles, which is capable of generating findings that place the process at the very top of the prediction system crop [33].

### Molecular Docking of Final Vaccine Epitopes With MHC Molecules

PEP-FOLD 2.0 from the RPBS Web Portal server [32] was used to predict the tertiary structure of the vaccine construct epitopes. PEP-FOLD is an online tool that was designed to model 3D protein conformation structures in aqueous solutions for proteins 9-25 amino acids in length (de novo modeling). PEP-FOLD conducts a series of 50 simulations beginning with an amino acid sequence, and returns the most critical energy and population-related conformations found [32].

The ClusPro 2.0 server [34,35] rotates the ligands of each of the final epitopes of a vaccine protein with 70,000 rotations. The ligand rotations are translated relative to the MHC receptor alleles in three axes (x, y, z) on a grid. The top 1000 lowest energy docked structures from 70,000 rotations are then chosen and processed in turn. This set might have the potential to consist of at least some models that are close to the native structure of the complex. The server then clusters the 1000 rotations by finding the structure with the most “neighbors” within a 9 Å interface root mean square deviation radius as the distance measure. This ligand and its neighbors are then considered as the “cluster center” and the “members” of the cluster, respectively. This process was repeated for the remainder of the ligands to find the next clusters. Finally, the server provides a score for the models and reports the top scoring models based on the cluster size (10 most populated clusters) [34,35]. One of the main advantages of ClusPro 2.0 as an automated protein docking server is its ability to generate protein-protein complexes with high accuracy [36].

PyMOL software was used to analyze the docking results. PyMOL is mostly utilized for molecular visualization by crystallographic, molecular dynamic simulation, and protein modeling software packages [37].

### Immune Response Simulation

IL4-, IL10-, and IFNγ-inducing proteins from the 8 epitopes in the final vaccine construct were predicted via IL4pred server [38], IL-10Pred server [39], and IFNepitope server [40], respectively.

The immune response to vaccine injection was simulated using the C-ImmSim 10.1 server [41]. C-ImmSim 10.1 is an agent-based computational immune-response simulator that utilizes a position-specific score matrix and machine-learning methods for predicting epitope and immune interactions, respectively [42]. We regulated the parameters based on the predominant human leukocyte antigen (HLA) alleles of predictions. The host HLA selection parameters for MHC class I were set to A1010, A1101, and B0702; the parameters for DR MHC class II were set to DBR1_0101; and the time step to injection was set to 1, 84, and 100 (maximum allowed value), respectively. We randomly shuffled the vaccine protein sequence (without adjuvants) using Stothard *P* 2000 from the Sequence Manipulation Suite server [43] to create a control group. The overall immunogenicity of the generic protein sequence associated with its amino acid sequence was assessed by this immune system simulation server [41]. The entire simulation was focused on three events, (1) B-cell epitopes binding, (2) HLA class I and II epitopes binding, and (3) T-cell receptor binding, in which the HLA–protein complex interaction should be present. Such processes are independently carried out by cells through various agents and the consumption of specific simulated biological quantities [41].

## Results

### Selection of Protein Sequences

The amino acid sequences of E protein and S protein of SARS-CoV-2 were collected from the NCBI virus database with accession numbers QHD43418 and QHR63280, respectively, which were released January 13, 2020, and have nucleotide completeness. The FASTA sequences were used to construct a multiepitope vaccine against SARS-CoV-2.

### B-Cell Epitopes Analysis

The ABCpred database reviewed the full-length E protein sequence for the analysis of linear B-cell epitopes. Among the results that passed the three filters of antigenicity, allergenicity, and toxicity, two epitopes (NVSLVKPSFYVYSRVK and YVYSRVKNLNSSRVPD) were chosen as protective epitopes (Table 1). The IEDB was then utilized to investigate the B-cell linear epitopes, resulting in 37 epitopes that were experimentally identified for S protein [44-54]. By contrast, there were no experimental B-cell epitopes for E protein of SARS-CoV-2.

### T-Cell Epitopes Analysis

The binding epitopes to MHC class II molecules of E protein were analyzed by the IEDB. We used the prediction method to identify T-cell epitopes of E protein since there were no corresponding experimental epitopes in this database, whereas we used the available experimental T-cell epitopes of S protein [55]. The same three filters of antigenicity, allergenicity, and toxicity were applied to identify the protective antigens. Based on the number of alleles, the predominant HLA alleles were HLA-DRA-DBR1*01:01 and HLA-DPB1*01:02 among MHC class II alleles.

### Antigenicity of Potential Epitopes

The antigenicity of both the B-cell and T-cell epitopes was predicted by VaxiJen 2.0, with a threshold of 0.4. The predicted epitopes with an antigenicity score above the threshold were considered as “antigen” epitopes. Screenings for the other two filters (allergenicity and toxicity) were not carried out on “nonantigen” epitopes (Table 1 and Table 2).

**Table 1:** Predicted T-cell and B-cell epitopes of SARS-CoV-2 envelope protein.^a^

Epitope			B-cell	MHC^b^ II	Antigenicity	Allergenicity	Toxicity
**ABCpred**							
	**Not selected for vaccine construction**						
		TLAILTALRLCAYCCN	+^c^	–^d^	Antigen	Nonallergen	Toxin
		LCAYCCNIVNVSLVKP	+	–	Antigen	Nonallergen	Toxin
		FVSEETGTLIVNSVLL	+	–	Nonantigen	Discontinued	Discontinued
	**Selected for vaccine construction**						
		NVSLVKPSFYVYSRVK	+	–	Antigen	Nonallergen	Nontoxin
		YVYSRVKNLNSSRVPD	+	+	Antigen	Nonallergen	Nontoxin
**IEDB^e^**							
	**Not selected for vaccine construction**						
		IVNSVLLFLAFVVFL	–	+	Antigen	Allergen	Discontinued
		EETGTLIVNSVLLFL	–	+	Antigen	Allergen	Discontinued
		GTLIVNSVLLFLAFV	–	+	Nonantigen	Discontinued	Discontinued
		IVNSVLLFLAFVVFL	–	+	Nonantigen	Discontinued	Discontinued
		MYSFVSEETGTLIVN	–	+	Nonantigen	Discontinued	Discontinued
		NIVNVSLVKPSFYVY	–	+	Antigen	Allergen	Discontinued
		RVKNLNSSRVPDLLV	–	+	Antigen	Allergen	Discontinued
		SEETGTLIVNSVLLF	–	+	Nonantigen	Discontinued	Discontinued
		TGTLIVNSVLLFLAF	–	+	Nonantigen	Discontinued	Discontinued
		YSFVSEETGTLIVNS	–	+	Nonantigen	Discontinued	Discontinued
	**Selected for vaccine construction**						
		SFYVYSRVKNLNSSR	–	+	Antigen	Nonallergen	Nontoxin
		FYVYSRVKNLNSSRV	–	+	Antigen	Nonallergen	Nontoxin
		YVYSRVKNLNSSRVP	–	+	Antigen	Nonallergen	Nontoxin
		FLAFVVFLLVTLAIL	–	+	Antigen	Nonallergen	Nontoxin
		FVSEETGTLIVNSVL	–	+	Antigen	Nonallergen	Nontoxin
		KPSFYVYSRVKNLNS	–	+	Antigen	Nonallergen	Nontoxin
		YSRVKNLNSSRVPDL	–	+	Antigen	Nonallergen	Nontoxin
		NSVLLFLAFVVFLLV	–	+	Antigen	Nonallergen	Nontoxin
		VKPSFYVYSRVKNLN	–	+	Antigen	Nonallergen	Nontoxin
		VNSVLLFLAFVVFLL	–	+	Antigen	Nonallergen	Nontoxin
		VSLVKPSFYVYSRVK	–	+	Antigen	Nonallergen	Nontoxin
		VVFLLVTLAILTALR	–	+	Antigen	Nonallergen	Nontoxin
		LLFLAFVVFLLVTLA	–	+	Antigen	Nonallergen	Nontoxin

^a^T-cell epitopes were identified as the best epitopes based on the number of alleles.

^b^MHC: major histocompatibility complex.

^c^+: Related.

^d^–: Unrelated.

^e^IEDB: Immune Epitope Database.

**Table 2:** Experimental T-cell and B-cell epitopes of SARS-CoV-2 spike protein from Immune Epitope Database.

Epitope	Selected for vaccine construction	B-cell	MHC^a^ II	Allergenicity	Toxicity
AATKMSECVLGQSKRVD	No	+^b^	–^c^	Allergen	Discontinued
CKFDEDDSEPVLKGVKLHYT	Yes	+	–	Nonallergen	Nontoxin
DDSEPVLKGVKLHYT	Yes	+	–	Nonallergen	Nontoxin
DKYFKNHTSPDVDLGD	Yes	+	–	Nonallergen	Nontoxin
DLGDISGINASVVNIQK	No	+	–	Allergen	Discontinued
EIDRLNEVAKNLNESLIDLQELGKYEQY	Yes	+	–	Nonallergen	Nontoxin
EVAKNLNESLIDLQELG	No	+	–	Allergen	Discontinued
KNHTSPDVDLGDISGIN	No	+	–	Allergen	Discontinued
LYQDVNC	No	+	–	Allergen	Discontinued
LYQDVNCT	No	+	–	Allergen	Discontinued
MAYRFNGIGVTQNVLY	Yes	+	–	Nonallergen	Nontoxin
MAYRFNGIGVTQNVLYE	Yes	+	–	Nonallergen	Nontoxin
RASANLAATKMSECVLG	No	+	–	Allergen	Discontinued
SPDVDLGDISGINAS	No	+	–	Allergen	Discontinued
MAYRFNGIGVTQNVLY	Yes	–	+	Nonallergen	Nontoxin
QLIRAAEIRASANLAATK	No	–	+	Allergen	Discontinued

^a^MHC: major histocompatibility complex.

^b^+: Related.

^c^–: Unrelated.

### Allergenicity of Potential Epitopes

The allergenicity of both B-cell and HTL epitopes was estimated by AllerTOP v. 2.0 (Table 1).

### Toxicity of Potential Epitopes

The toxicity of both B-cell and HTL epitopes was predicted by ToxinPred, with a protein fragment length of 10 (Tables 1 and 2).

### Construction of the Chimeric Protein

The screened epitopes were chosen for the design of a chimeric protein as a multiepitope vaccine. As shown in Tables 1 and 2, we selected 21 epitopes (including 6 B-cell epitopes and 1 HTL epitope of S protein, and 2 B-cell epitopes and 12 HTL epitopes of E protein), which all filled the criteria of “antigen,” “nonallergen,” and “nontoxin.” To establish a contiguous sequence in the final construction, the overlapping sequences of B-cell and T-cell epitopes were merged. In detail, MAYRFNGIGVTQNVLYE was obtained from MAYRFNGIGVTQNVLY (S protein HTL epitope), MAYRFNGIGVTQNVLY, and MAYRFNGIGVTQNVLYE (S protein B-cell epitopes); CKFDEDDSEPVLKGVKLHYT was obtained from CKFDEDDSEPVLKGVKLHYT and DDSEPVLKGVKLHYT (S protein B-cell epitopes); SFYVYSRVKNLNSSRVPDL was obtained from YVYSRVKNLNSSRVPD (E protein B-cell epitope), SFYVYSRVKNLNSSR, YVYSRVKNLNSSRVP, and YSRVKNLNSSRVPDL (E protein HTL epitopes); VNSVLLFLAFVVFLLVTLAILTALR was obtained from VVFLLVTLAILTALR, LLFLAFVVFLLVTLAILTA, FLAFVVFLLVTLAIL, NSVLLFLAFVVFLLV, and VNSVLLFLAFVVFLL (E protein HTL epitopes); and NVSLVKPSFYVYSRVKNLNS was obtained from NVSLVKPSFYVYSRVK (E protein B-cell epitope), VSLVKPSFYVYSRVK, VKPSFYVYSRVKNLN, and KPSFYVYSRVKNLNS (E protein HTL epitopes). Predicted linear B-cell epitopes and T-cell epitopes were connected utilizing KK linkers as flexible connectors (Figure 1B).

The arrangement of epitopes in the final vaccine construct had a substantial effect on the physicochemical properties such as half-life and instability, with the half-life varying from 5.5 hours to 30 hours in mammalian cells, and the stability varying from an unstable to a completely stable protein simply by changing the order of epitopes. Therefore, we further investigated the properties of more than 40 possible permutations considering the overlaps of the selected epitopes to find the best formulation of this vaccine candidate.

### Antigenicity, Allergenicity, and Toxicity Estimation of the Candidate Multiepitope Vaccine

The antigenicity of the final protein chimera (Figure 1B) was estimated by the VaxiJen 2.0 server to be 0.5830 with a threshold of 0.4. The ANTIGENpro platform was also utilized to estimate the antigenicity of the final protein. Based on this server, the whole protein (Figure 1B) is predicted as an antigen with a probability of 0.415508. The AllerTOP v.2.0 server indicated that the final protein is predicted as a “nonallergen” and the ToxinPred server predicted the final protein as a “nontoxin.”

### Amino Acid Composition, Physicochemical Properties, and Solubility Prediction

Based on the Protparam database, the final protein chimera comprised 173 amino acids (Figure 1B) with a molecular weight of 19.9 kDa. The pI value was predicted to be 9.57. The half-life was estimated to be 30 hours in mammalian reticulocytes in vitro, more than 20 hours in yeast, and over 10 hours in *E. coli* in vivo. Instability index II was predicted to be 26.45, classifying the protein as stable (an index>40 indicates instability). The aliphatic index was 101.21, indicating high thermostability. The estimated GRAVY value was –0.293. This negative attribute indicates that the protein is hydrophilic and can react with water molecules. Furthermore, based on the PepCalc server, the solubility was predicted to be “good” in water. Based on SOLpro from ANTIGENpro, the protein chimera was expected to be soluble with a probability of 0.765767.

### Secondary Structure Prediction

**Figure 2:**
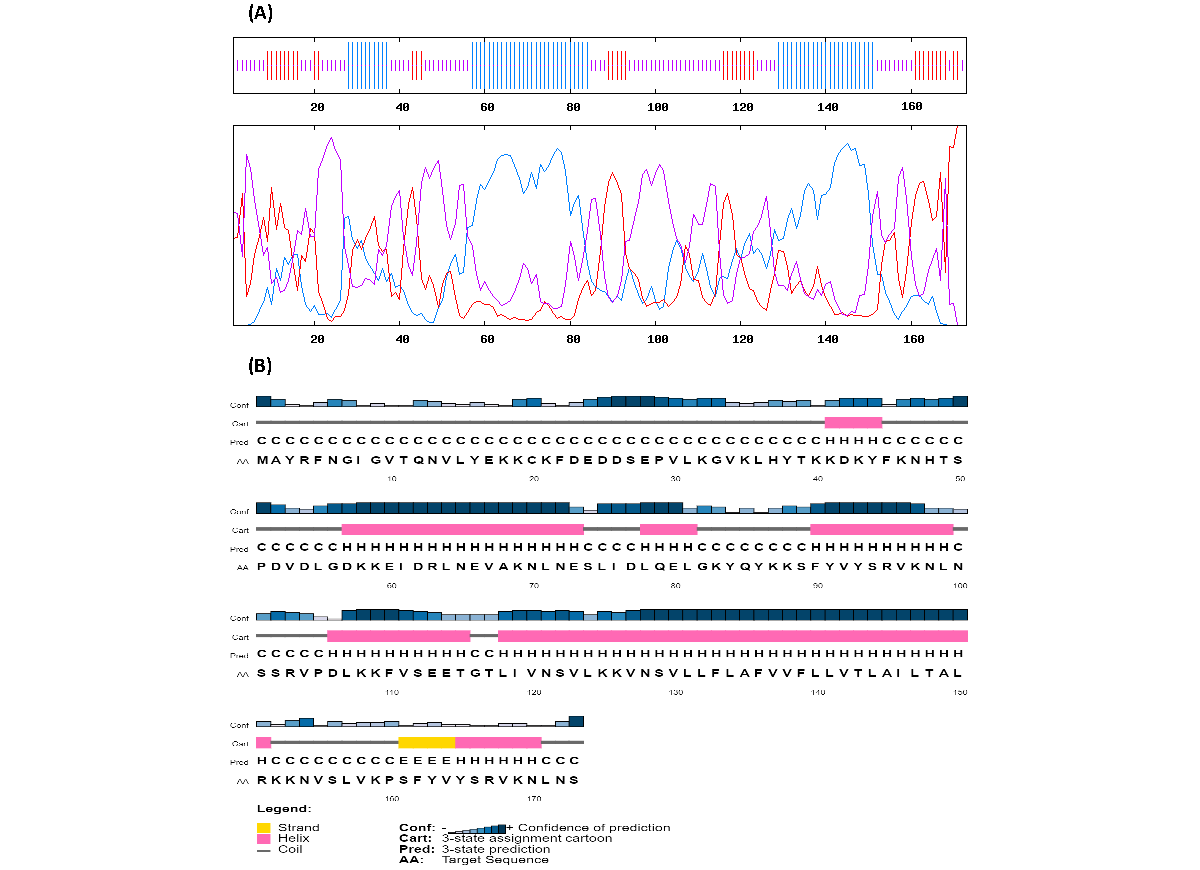
Properties of the final vaccine protein candidate. (A) Secondary structure of the final protein predicted by the Prabi server. Red: extended strands; blue: alpha helix configurations; purple: random coils. (B) The residues and their arrangements analyzed by the PSIPRED 4.0 server. Yellow and pink regions indicate strands and helices, respectively; gray linkers indicate coil configurations.

The secondary structure of the final protein (Figure 1B) was analyzed by the Prabi server. The final chimeric protein was estimated to include 35.26% alpha helices, 20.81% extended strands, and 43.93% random coils (Figure 2A). The detailed secondary structure predicted by the PSIPRED 4.0 server, including the residues and their configurations, is shown in Figure 2B.

### Molecular Docking of Final Epitopes of the Vaccine With MHC Molecules

**Figure 3:**
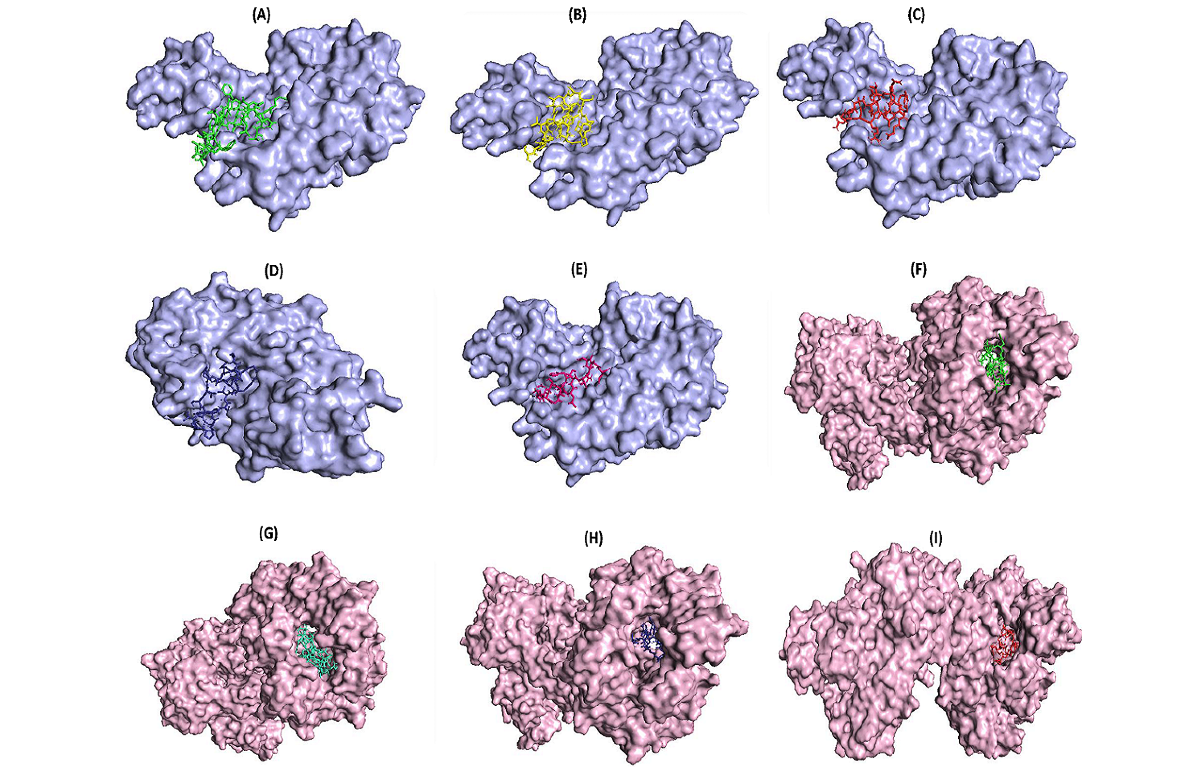
Molecular docking analysis. (A) VNSVLLFLAFVVFLLVTLAILTALR epitope (green) and HLA-DPA1*01:03 protein (blue). (B) SFYVYSRVKNLNSSRVPDL epitope (yellow) and HLA-DPA1*01:03 protein (blue). (C) MAYRFNGIGVTQNVLYE epitope (red) and HLA-DPA1*01:03 protein (blue). (D) NVSLVKPSFYVYSRVKNLNS epitope (dark blue) and HLA-DPA1*01:03 protein (blue). (E) FVSEETGTLIVNSVL epitope (pink) and HLA-DPA1*01:03 protein (blue). (F) VNSVLLFLAFVVFLLVTLAILTALR epitope (green) and HLA-DRB1*01:01 protein (light pink). (G) EIDRLNEVAKNLNESLIDLQELGKYQY epitope (light blue) and HLA-DRB1*01:01 protein (light pink). (H) NVSLVKPSFYVYSRVKNLNS epitope (dark blue) and HLA-DRB1*01:01 protein (light pink). (I) MAYRFNGIGVTQNVLYE epitope (red) and HLA-DRB1*01:01 protein (light pink). HLA: human leukocyte antigen.

The crystal structures of HLA-DRA-DBR1*01:01 and HLA-DPB1*01:02 were retrieved from the PDB RCSB database (PDB ID: 1AQD and 3LQZ, respectively). The PDB files were edited and cleaned from heteroatoms. PEP-FOLD 2.0 from the RPBS web portal server was used to predict the tertiary structure of 8 epitopes of the vaccine construct individually. Molecular docking was performed on the epitopes and the whole vaccine construct with relevant MHC alleles using the ClusPro 2.0 online server. PyMOL software was used to perform a detailed analysis of the interface of protein-protein interactions (Figure 3). The weighted score of the lowest energy docked complexes are reported in Table 3. The best way to rank the model is according to the cluster size (number of members) [34,35]. The most populated clusters were found in MAYRFNGIGVTQNVLYE and HLA-DPA1*01:03, DKYFKNHTSPDVDLGD and HLA-DPA1*01:03, CKFDEDDSEPVLKGVKLHYT and HLA-DPA1*01:03, and EIDRLNEVAKNLNESLIDLQELGKYQY and HLA-DRB1*01:01, with 784, 577, 350, and 261 cluster members, respectively.

**Table 3:** Docking results and prediction of the immunity effects of epitopes.

Vaccine epitopes	Weighted scores^a^ of the complex docked with				
	HLA^b^-DPA1*01:03	HLA-DRB1*01:01	IL^c^4 inducer	IL10 inducer	IFN^d^γ inducer
MAYRFNGIGVTQNVLYE	–869.2	–962.2	–^e^	–	–
CKFDEDDSEPVLKGVKLHYT	–758.7	–810.6	+^f^	–	+
DKYFKNHTSPDVDLGD	–763.2	–755	+	–	–
EIDRLNEVAKNLNESLIDLQELGKYQY	–715.4	–1050.7	–	+	+
SFYVYSRVKNLNSSRVPDL	–875.5	–992.2	+	+	+
FVSEETGTLIVNSVL	–798.2	–823.9	+	+	–
VNSVLLFLAFVVFLLVTLAILTALR	–1148.3	–1477	–	+	+
NVSLVKPSFYVYSRVKNLNS	–852.7	–1036.6	+	+	+

^a^The weighted scores of the lowest energy docked structures were based on the cluster size of the most populated cluster.

^b^HLA: human leukocyte antigen.

^c^IL: interleukin.

^d^IFN: interferon.

^e^–: Unrelated.

^f^+: Related.

### Immune Response Simulation

We predicted the IL4, IL10, and IFNγ inducing proteins from the 6 epitopes in the final vaccine construct via IL4pred server, IL10pred server, and IFNepitope server, respectively. The results are shown in Table 3.

**Figure 4:**
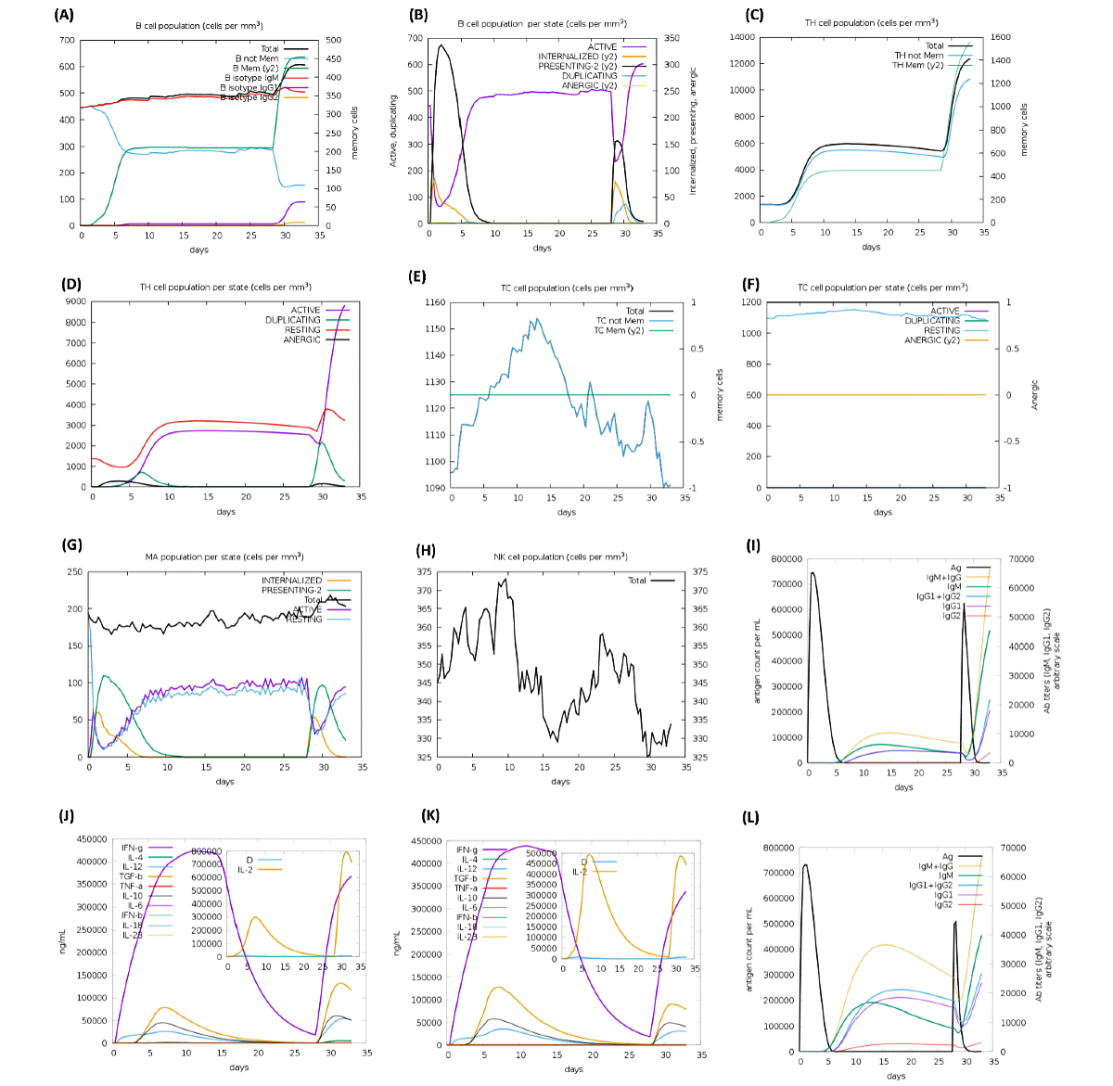
In silico immune response simulation to the injection of the candidate vaccine and control protein by the C-ImmSim 10.1 server. The simulation was performed with three injections in the time steps of 1, 84, and 100; each time step is equal to 8 hours. (A) B-cell population. (B) B-cell population per state. (C) T helper (TH) cell population. (D) TH cell population per state. (E) T cytotoxic (TC) cell population. (F) TC cell population per state. (G) Macrophage (MA) cell population. (H) Natural killer (NK) cell population. (I) Immunoglobulins. (J) Cytokines. (K) Cytokines after the protein control injection. (L) Immunoglobulins following the protein control injection.

**Figure 5:**
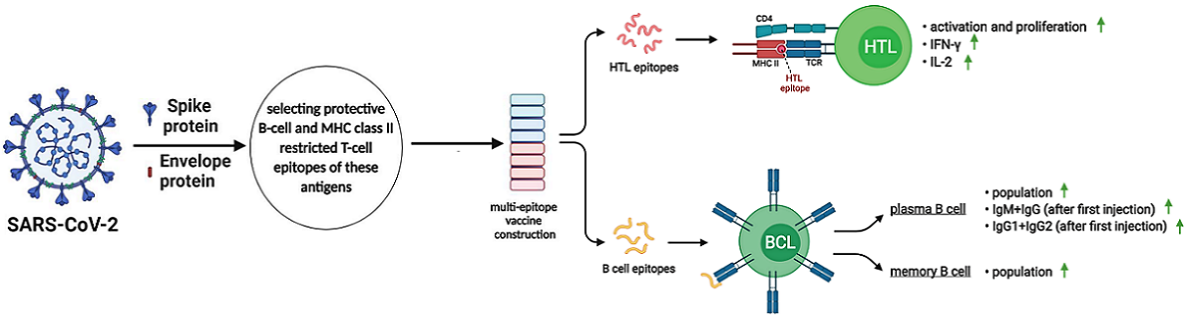
Graphical representation of immune response simulation to the injection of the vaccine candidate against SARS-CoV-2 spike (S) and envelope (E) proteins. HTL: helper T lymphocyte; BCL: B-cell lymphoma; IgM: immunoglobulin M; IgG: immunoglobulin G; IFN-γ: interferon gamma; IL-2: interleukin-2; TCR: T-cell receptor; MHC class II: major histocompatibility complex class II; CD4: cluster of differentiation 4. The image was created using the Biorender illustrator tool.

The primary and secondary immune responses were stimulated by the C-ImmSim 10.1 server. This server simulated the immune response of vaccine candidates with three injections in the time steps of 1, 84, and 100; each time step is equal to 8 hours. To perform a relative comparison, we created a shuffled sequence of the vaccine candidate as a control protein, and we analyzed the results of the immune response simulation to the injection of the control. This shuffled sequence was employed to evaluate the significance of the vaccine sequence results, because in immune response simulation by this server, the sequence composition of the final epitopes connected via KK linkers is an important consideration. The results of the vaccine injection clearly varied from those of the controls (Figure 4 and Figure 5).

## Discussion

### Principal Findings

EVs offer a new strategy for the prophylactic and therapeutic use of pathogen-specific immunity [56]. A multiepitope vaccine consisting of a protein series or overlapping proteins has been proposed as an appropriate solution to the prevention and treatment of viral infections [57-62]. The perfect multiepitope vaccine should be engineered to include epitopes that can activate cytotoxic T lymphocytes, T-cells, and B-cells, and trigger successful responses to specific viruses [57].

We here present the in silico design of a potential multiepitope vaccine against the S and E proteins of SARS-CoV-2, which comprises both B-cell and HTL epitopes and can stimulate the immune system responses impressively. Immune interference is less likely to be a concern for multicomponent vaccines against a specific organism. For multitarget vaccinations, a strong response to one immune agent may reduce the otherwise marginal reaction to the second immunogen, and thus render the individual susceptible to infection with the pathogen corresponding to the second immune agent [63]. Since the SARS-CoV-2 S glycoprotein is surface-exposed and facilitates entry into host cells, it is the major priority of neutralizing antibodies against infection and the target of therapeutic and vaccine development [64,65]. S protein is also a primary focus for the design of subunit vaccines for SARS-CoV and Middle East Respiratory Syndrome (MERS)-CoV [66]. S trimers are widely coated with N-linked glycans, which are crucial for efficient folding and for modulating accessibility to host proteases and neutralizing antibodies [65-70]. E protein is conserved in all coronaviruses and covers the entire surface of SARS-CoV-2 (Figure 1A). There are fewer toxic epitopes of E protein than found for S protein. This finding was verified in the literature, in which E protein was explored in SARS-CoV in 2003 and, more recently, in MERS-CoV, demonstrating the retention of this protein in seven strains using the BioEdit Package tool and less toxic regions than in the S protein [12,57-62]. Several studies have examined the potential of coronaviruses with mutated E protein, focusing specifically on SARS- and MERS-CoV, as live attenuated vaccine candidates associated with hopeful results [12,71-75]. We obtained the FASTA sequence of the S and E proteins of SARS-CoV-2 from the NCBI database. B-cell and HTL epitopes of E protein were predicted by different servers, whereas experimentally confirmed epitopes were utilized for S protein. The epitopes were screened based on the three filters of antigenicity, allergenicity, and toxicity. Therefore, we selected only protective epitopes. We merged the overlaps of B-cell and T-cell epitopes and fused them with appropriate flexible linkers. Previous studies reported that KK linkers preserve independent immune responses when they are inserted between epitopes [26] (Figure 1B).

The absence of allergenic properties of the proposed protein chimera further increases its potential as a vaccine candidate [76]. Finally, the whole-protein chimera was analyzed for antigenicity, allergenicity, and toxicity, which was predicted as an antigen [22], nonallergen [20], and nontoxin [25]. The pI was calculated to be 9.57, which shows that the final protein is alkaline. The vaccine protein construct was predicted as “soluble” upon expression in the *E. coli* host. The structural stability of a vaccine is known to be an essential aspect of its effectiveness, which can ensure the appropriate presentation of antigens and thus efficiently activate the immune system [77,78]. The instability index II of our candidate was calculated to be 26.45, which indicates that this protein is “stable.”

Secondary structure analysis predicted that the final protein consists of 35.26% alpha-helices, 20.81% extended strands, and 43.93% random coils. Essential types of “structural antigens” have been identified as natively unfolded protein regions and alpha-helical coil proteins. These two structural types, when examined in synthetic proteins, can fold into their native structure and are therefore recognized by antibodies naturally triggered in response to infection [76,79]. In the context of structural vaccinology, a molecular docking study was needed to predict the binding affinity of epitopes to the crystallized fragment of antibodies or MHC molecules [80,81]. To analyze the affinity of the final multiepitope vaccine to MHC molecules, we performed 16 docking simulations on the 8 epitopes of the final vaccine with MHC class II receptors. The results of docking analyses were notable, demonstrating the high affinity of the final epitopes of the vaccine construct to MHC molecules. The interface of protein-protein interactions was further considered using a visualization tool.

In the next step of designing a multiepitope vaccine, a systems vaccinology approach is beneficial in assessing the human complex immune response at different stages of biological structures [82]. Finally, we utilized an immune simulator server to predict the primary and secondary responses of the immune system to three injections of the candidate vaccine. From the cytokines simulation plot, we noted an increase in the levels of IL-4 and IFNγ, which is similar to the clinical features of COVID-19 patients reported by Huang et al [2] (Figure 4J). Appropriate activation of APCs, high production of memory cells due to the extensive activation of B-cells and T-cells, control and clearance of antigens due to the creation of cytokines by the participation of T helper memory cells, and the evident long-term memory persistence after three injections could confirm the efficiency of our candidate vaccine [83].

Finally, we selected one of the multiepitope vaccine candidates with the lowest molecular weight (shortest sequence length), which can potentially result in low-cost manufacturing and shorten production times [84].

### Comparison With Prior Work

Unlike most of the multiepitope vaccines that have been suggested during the COVID-19 pandemic, we preferred to design a vaccine without built-in adjuvants. Since adjuvants are necessary to increase the dosage efficacy by preventing the rapid degradation of proteins [85], tank-mixed adjuvants can be added to the final formulation. For instance, aluminum salts can be candidate adjuvants as they are used in various viral and bacterial vaccines and would be expected to enhance the antigen stability [86].

Adjuvants are effective in vaccine stability and can contribute to immunization reduction and enhanced antibody responses. Although our suggested vaccine lacks a built-in adjuvant, it demonstrates the same stability and half-life estimation as previously reported candidates. Our vaccine construct also showed roughly the highest AI in comparison with other suggested multiepitope vaccines, along with the highest thermostability [29]. These benefits are only achieved by the careful arrangement of selected epitopes in designing this vaccine construct. This study thus demonstrates the importance of testing various permutations of epitopes in vaccine properties.

Finally, discharging our multiepitope vaccine from built-in adjuvants can demonstrate that the immune simulation for two injections (Figure 4) was induced because of the designed vaccine without interference from any nonspecific immunization against the adjuvants.

### Conclusion

The goal of this research was to suggest a computational method for predicting protective B-cell and T-cell epitopes of the E protein of SARS-CoV-2 accompanied by experimental epitopes of S protein to construct a chimeric protein vaccine candidate against this pandemic disease. The results demonstrated the high affinity of this chimeric protein to MHC molecules of the immune system, and the outputs of immune response simulation to the injection of this novel vaccine confirmed our findings. Thus, this multiepitope vaccine designed against the S and E proteins of SARS-CoV-2 utilizing immunoinformatics methods may be considered a new, safe, and efficient approach against SARS-CoV-2.

## Abbreviations

ACC: auto cross covariance
APC: antigen-presenting cell
E protein: envelope protein
EV: epitope-based vaccine
FN: false negative
FP: false positive
GRAVY: grand hydropathic average
HLA: human leukocyte antigen
HTL: helper T lymphocyte
IEDB: Immune Epitope Database
IFN: interferon
IL: interleukin
IP10: interferon-inducible protein 10
KK: bilysine
MCP1: monocyte chemoattractant protein 1
MERS: Middle East respiratory syndrome
MHC: major histocompatibility complex
M protein: membrane protein
NCBI: National Center for Biotechnology Information
N protein: nucleocapsid protein
pI: isoelectric point
S protein: spike protein
TN: true negative
TP: true positive
